# A novel hybrid GWO–PSO-based maximum power point tracking for photovoltaic systems operating under partial shading conditions

**DOI:** 10.1038/s41598-022-14733-6

**Published:** 2022-06-23

**Authors:** Smail Chtita, Saad Motahhir, Aboubakr El Hammoumi, Aissa Chouder, Abou Soufiane Benyoucef, Abdelaziz El Ghzizal, Aziz Derouich, Mohamed Abouhawwash, S. S. Askar

**Affiliations:** 1 Industrial Technologies and Services Laboratory, EST, SMBA University, Fez, Morocco; 2 Engineering, Systems and Applications Laboratory, ENSA, SMBA University, Fez, Morocco; 3 Innovative Technologies Laboratory, EST, SMBA University, Fez, Morocco; 4 Electrical Engineering Laboratory (LGE), University Mohamed Boudiaf of M’sila, BP 166, 28000 M’Sila, Algeria; 5 University Djilali Bounaama, Khemis Miliana, Rue Thniat Elhad, Khemis Miliana, Algeria; 6 Department of Mathematics, Faculty of Science, Mansoura University, Mansoura, 35516 Egypt; 7 Department of Computational Mathematics, Science, and Engineering (CMSE), College of Engineering, Michigan State University, East Lansing, MI 48824 USA; 8 Department of Statistics and Operations Research, College of Science, King Saud University, Riyadh, 11451 Saudi Arabia

**Keywords:** Energy science and technology, Renewable energy, Solar cells

## Abstract

One of the major challenges in photovoltaic (PV) systems is extracting the maximum power from the PV array, especially when they operate under partial shading conditions (PSCs). To address this challenge, this paper introduces a novel hybrid maximum power point tracking (MPPT) method based on grey wolf optimization and particle swarm optimization (GWO–PSO) techniques. The developed MPPT technique not only avoids the common disadvantages of conventional MPPT techniques (such as perturb and observe (P&O) and incremental conductance) but also provides a simple and robust MPPT scheme to effectively handle partial shading in PV systems, since it requires only two control parameters, and its convergence to the global maximum power point (GMPP) is independent of the search process's initial conditions. The feasibility and effectiveness of the hybrid GWO–PSO-based MPPT method are verified via a co-simulation technique that combines MATLAB/SIMULINK and PSIM software environments, while comparing its performance against GWO, PSO and P&O based MPPT methods. The simulation results carried out under dynamic environmental conditions have shown the satisfactory effectiveness of the hybrid MPPT method in terms of tracking accuracy, convergence speed to GMPP and efficiency, compared to other methods.

## Introduction

Photovoltaic (PV) technology has gained more and more maturity in the last two decades and has been being deployed massively worldwide as a secured and reliable source of energy. Despite the significant costs’ reduction of the PV systems components, the energy harvesting optimization is still an important issue to make them cost and efficiency competitive^1^. This is the main reason why these systems must include maximum power point tracking (MPPT) controllers^2^. However, the achievement of MPPT remains a challenging task due to the dynamic behaviour of the PV array, which is significantly impacted by environmental conditions. This challenge becomes even more convoluted in the case of partial shading conditions (PSCs), which occur when the entire PV array is not receiving homogeneous insolation, which can result from several factors, like clouds, trees, buildings or even dust^3^. Furthermore, under PSCs, the PV array’s power-voltage (P–V) curve exhibits several power maxima, which result from the use of bypass diodes that protect shaded PV cells from damage caused by the hot-spot phenomenon^4,5^.

Due to their simplicity and effectiveness in tracking the maximum power point (MPP) under homogeneous insolation conditions, traditional MPPT methods such as hill-climbing, perturb and observe (P&O), and incremental conductance (INC) are the most commonly used in various types of PV systems such as stand-alone PV systems^6^, solar pumping systems^7^ and grid-connected PV systems^8^. However, under PSCs, these conventional MPPT techniques do not differentiate between a global MPP (GMPP) and a local MPP (LMPP), since they converge to the MPP that comes in contact first, which is most likely a local MPP^9^. Therefore, this leads to a considerable energy loss of up to 70%^10^, far away from the desired optimal energy harvesting.

Alternatively, many enhancements of conventional MPPT methods have been made to handle partial shading in PV systems, which can be categorized into topology-based and algorithm-based MPPT methods. The MPPT methods based on topology need extra electrical circuits to carry out the global MPPT (GMPPT)^11,12^, which consequently reduces the overall system efficiency and also increases the total cost. While the algorithm-based MPPT methods, such as fuzzy logic with polar information controller^13^, dividing rectangles (DIRECT) search control^14^ and sequential extremum seeking control^15^, have some disadvantages, such as high cost, extensive computational effort and greater complexity of hardware implementation.

Recently, nature-inspired optimization algorithms have become popular due to their effectiveness in dealing with complex problems^16^, such as the non-linear behaviour of PV array. In addition, their ease of implementation makes them attractive to address the MPPT issue in PV systems operating under PSCs. Among the nature-inspired optimization algorithms extensively employed in MPPT techniques are evolutionary algorithms (EA) such as differential evolution (DE)^17^ and genetic algorithm (GA)^18^. However, these algorithms are mainly based on a trial-and-error process for parameter setting, which results in a significant computational time^19^.

In turn, swarm intelligence (SI) based optimization algorithms like particle swarm optimization (PSO)^20–22^ and ant colony optimization (ACO)^23^ have also been employed in the design of MPPT controllers. Although these algorithms (PSO and ACO) offer important advantages, such as reduced computational effort and independence from internal system parameters, they require the determination of three parameters, which renders these algorithms inflexible. Moreover, PSO convergence is heavily dependent on the agents' initial position.

Artificial bee colony (ABC)^24,25^ and grey wolf optimization (GWO)^26^ are relatively new members of SI techniques, which have also been used in many research works to address with partial shading issues in PV systems. Their findings indicate the simplicity and flexibility of both algorithms since they only require two control parameters and their convergences to GMPP are not dependent on the search process’s initial conditions. Compared to the PSO algorithm, the ABC algorithm suffers from a major drawback in terms of the slow convergence to GMPP^22^. On the other hand, some hybrid techniques, such as GWO assisted P&O (GWO-P&O)^27^, and GWO with fuzzy logic controller (GWO-FLC)^28^, have been proposed in the literature to boost the effectiveness of the GWO algorithm to handle partial shading in PV systems. Although these hybrid techniques give better outcomes than the original method, the designed MPPT controllers are sophisticated and time-consuming.

In 2017, Narinder Singh et al. proposed the hybrid algorithm of GWO and PSO (GWO–PSO) as a novel member of SI techniques^29^. This algorithm combines GWO's exploration ability with PSO's exploitation ability to produce the strength of both variants. Toward this end, the authors used a mixed, low-level and co-evolutionary hybridization. Mixed because it involves two distinct variants for the global optimal solution generation, low-level because it merges both variants' functionalities, and co-evolutionary because both variants run in parallel. Based on all these modifications, the hybrid GWO–PSO algorithm showed highly competitive results in terms of computational simplicity, convergence independent of initial conditions, high solution accuracy, and capacity to cope with local minima compared to famous SI-based algorithms^29^. Nonetheless, to the authors' knowledge, there is no reference in the literature that deals with the hybrid GWO–PSO algorithm for MPPT applications in PV systems. Accordingly, this paper aims to propose a new hybrid GWO–PSO algorithm dedicated to PV systems operating under PSCs. In this algorithm, GWO search agents extensively explore the search space to avoid LMPPs, and thus can converge towards GMPP. This exploration is controlled by the PSO algorithm, which in turn improves the obtained solutions progressively during the process in order to accelerate the convergence towards GMPP in the exploitation phase. The effect of combining these two algorithms (GWO and PSO) allows reaching GMPP in only a few steps, thus making the algorithm more efficient. In addition, the MPPT controller developed in this work adopts a direct control technique, which means that the GWO–PSO algorithm directly adjusts the duty cycle without the need to employ a linear compensator, simplifying the process and eliminating any computational burden associated with compensator gains adjustment. Moreover, the proposed MPPT scheme can be considered as an attractive opportunity for PV systems operating under both homogeneous and inhomogeneous insolation conditions due to these benefits:

- Excellent tracking ability with high accuracy.
- Convergence to GMPP independent of the search process's initial conditions.
- The use of only two control parameters for adjustment.
- No prior knowledge of the PV modules' characteristics is required.

After the introduction, the next section describes the PV array configuration studied in this work, including P–V characteristics of the used shading patterns. Section “Proposed hybrid GWO–PSO algorithm applied to MPPT control” details the working principle of the hybrid GWO–PSO algorithm, as well as its application for the MPPT controller. The simulation results of the proposed GWO–PSO based MPPT method, including a comparison of its performance against GWO, PSO, and P&O based MPPT methods, are given and discussed in Section “Implementation and results”. Lastly, Section “Conclusion” summarizes the main results of this investigation.

### System description under PSCs

**Figure 1:**
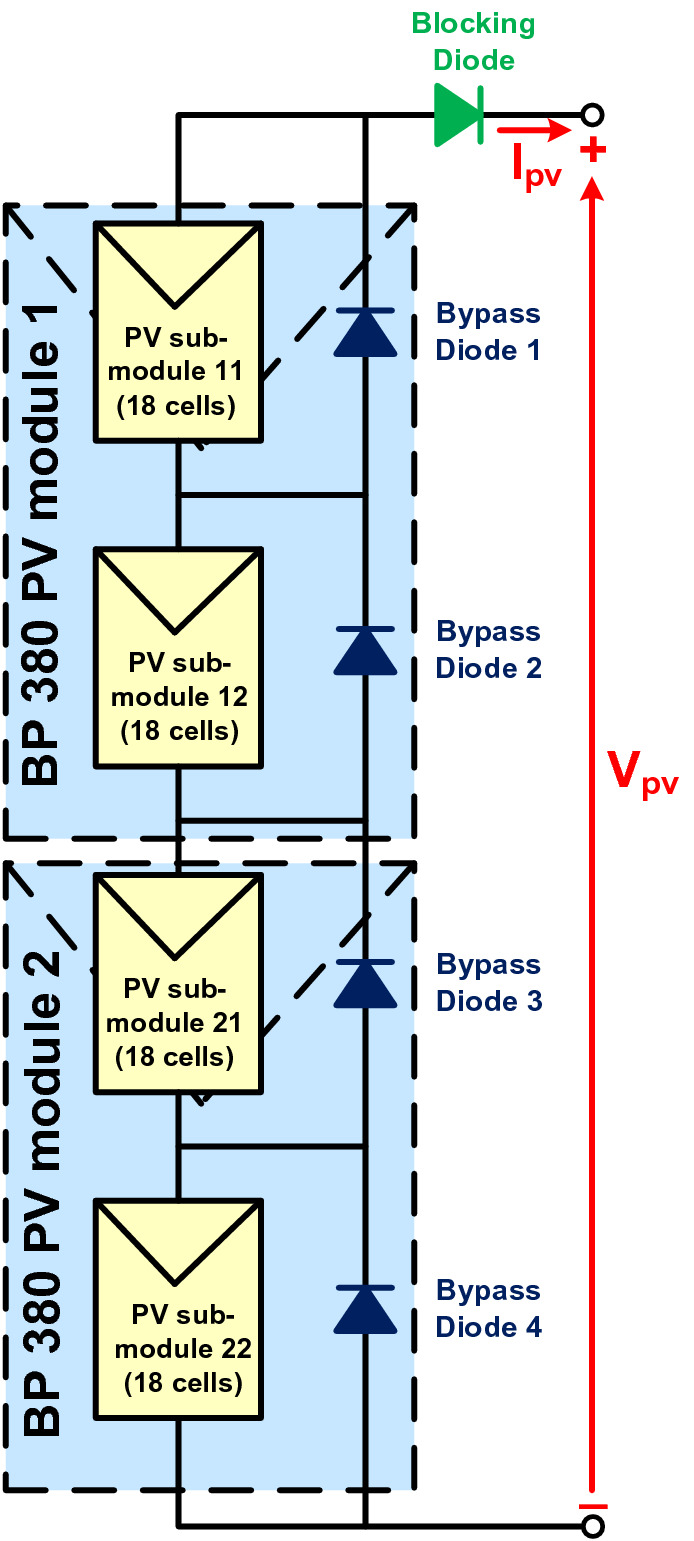
PV array configuration using two BP 380 PV modules serially connected.

**Figure 2:**
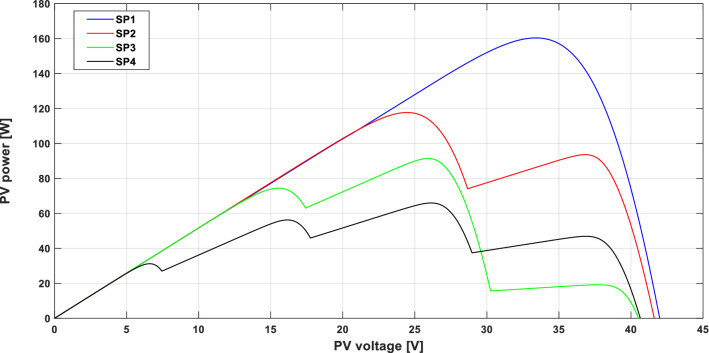
P–V characteristics of used patterns.

A typical PV array comprises multiple PV modules wired in series and/or parallel to produce the appropriate current and voltage for the load. To protect shaded PV modules from hot-spot damage caused by PSCs, blocking diodes and bypass diodes are used^4^. In this work, a PV array composed of two BP 380 PV modules serially connected is used, where two bypass diodes are considered to protect eighteen cells in each PV module (i.e., a 2S1P configuration) as illustrated in Fig. 1. Table 1 presents the specifications of the PV module used^30^ and Table 2 shows the four different shading patterns (SPs) that are considered in this study; the corresponding P–V curve of each SP is given in Fig. 2. For SP1, all PV sub-modules receive the same insolation, and the bypass diodes are, therefore, reverse biased and do not exhibit any effect. Hence, the current passes through each PV module and thus the PV array's P–V curve exhibits only one peak as depicted in Fig. 2. This situation is known as uniform insolation. In contrast, for patterns SP2, SP3 and SP4, the PV sub-modules do not receive the same insolation (i.e., case of PSC), the bypass diodes across the shaded PV sub-modules are, therefore, forward biased. Hence, the current from the unshaded PV sub-modules passes through the bypass diodes instead of the shaded PV sub-modules, to avoid damaging the shaded PV modules. In this case, multiple peaks appear in the PV array's P–V curve with only one GMPP, as clearly illustrated in Fig. 2. Accordingly, the PV array should operate continuously at the GMPP to extract the maximum instantaneous PV power under PSCs, thus avoiding a power loss of up to 70%^10,31^.

**Table 1:** BP 380 PV module specifications at STC.

Characteristics	Value
Maximum power	80 W_p_
Maximum power voltage	17.6 V
Maximum power current	4.55A
Short circuit current	4.8A
Open-circuit voltage	22.1 V
Current temperature coefficient	0.00312 A/°C
Voltage temperature coefficient	− 0.080 V/°C
No. of cells	36

**Table 2:** The four shading patterns used in this study.

Pattern no.	Insolation on PV sub-modules (W/m^2^)			
	Gpv11	Gpv12	Gpv21	Gpv22
SP1	1000	1000	1000	1000
SP2	1000	500	1000	1000
SP3	1000	700	100	1000
SP4	1000	700	500	250

Given all the above, an intelligent and efficient MPPT method is required to harvest the available optimal power from the PV array under PSCs.

## Proposed hybrid GWO–PSO algorithm applied to MPPT control

### Mathematical modelling

#### PSO algorithm

PSO is a nature-inspired metaheuristic algorithm, firstly introduced by Kennedy et al. in 1995^32^. It was modelled primarily by the simulation of the foraging behaviour of bird flocks. Based on swarm intelligence, the PSO algorithm manages a number of cooperative particles to explore the entire search space. Each particle has a unique position, $x_{i}$, and velocity, $v_{i}$, which could represent a candidate solution. During the search process, a particle's position is influenced by a particle's best position in a neighbourhood, $P_{best,i}$, and by the best position of all particles in the whole population, $G_{best}$. Accordingly, the particle position, $x_{i}$, is updated using the following equation:

1 $$x_{i}^{{k{ + }1}} = x_{i}^{k} { + } v_{i}^{{k{ + }1}}$$

where $v_{i}$ is the particle velocity which is computed by the following equation:

2 $$v_{i}^{{k{ + }1}} = w v_{i}^{k} { + }c_{1} r_{1} \left( {P_{best,i} { - }x_{i}^{k} } \right){ + }c_{2} r_{2} \left( {G_{best} { - }x_{i}^{k} } \right)$$

where $k$ is the number of iterations, $c_{1}$ and $c_{2}$ are the acceleration coefficients, $r_{1}$ and $r_{2}$ denote uniformly distributed random variables within the interval [0, 1], and $w$ represents the inertia weight.

#### GWO algorithm

GWO is a novel member of SI-based metaheuristic algorithms firstly developed by Mirjalili et al. in 2014^33^. It stimulates the grey wolves' social behaviour and mimics their leadership hierarchy and hunting process in nature. In a pack, grey wolves possess an extremely strict dominant social hierarchy, consisting of four levels. The leaders, which are both female and male, are named alpha (α). The subordinate wolves, which assist the leaders, are known as beta (β) and represent the second level of the grey wolves' hierarchy. The third level of this hierarchy is called delta (δ), while the remaining wolves are termed omega (ω) and represent the lowest level of the hierarchy. In this latter, the grey wolves' dominance increases from alpha (α) to omega (ω).

The GWO algorithm divides the candidate solutions into four groups for modelling the leadership hierarchy: alpha is the best solution, beta is the second-best solution, delta is the third best solution, and omega represents the rest of the solutions. This algorithm's solution generation process is divided into three stages:

##### Encircling prey

This operation represents the first stage of the hunt, where the grey wolves start encircling the prey. The mathematical modelling of this stage is described as follows:

3 $$\overrightarrow {D} = \left| {\overrightarrow {C} \overrightarrow {X}_{P} (t) - \overrightarrow {X} (t)} \right|$$

4 $$\overrightarrow {X} (t + 1) = \overrightarrow {X}_{P} (t) - \overrightarrow {A} \overrightarrow {D}$$

where $\overrightarrow {X}$ and $\overrightarrow {X}_{P}$ represent respectively the position vector of a search agent (wolf position) and the position vector of the optimal solution (prey position), and $t$ is the current iteration. The coefficient vectors, denoted by $\overrightarrow {A}$ and $\overrightarrow {C}$, are computed as follows:

5 $$\overrightarrow {A} = 2\overrightarrow {a} \overrightarrow {r}_{1} - \overrightarrow {a}$$

6 $$\overrightarrow {C} = 2\overrightarrow {r}_{2}$$

where components of $\overrightarrow {a}$ are linearly decreased from 2 to 0 during iterations, and $\overrightarrow {r}_{1}$, $\overrightarrow {r}_{2}$ are random vectors in the interval [0, 1].

##### Hunting

This operation is directed by alpha (α) (which represents the best candidate solution), beta (β) and delta (δ), as they have greater knowledge of the likely location of the optimal solution (i.e., the prey). The remaining search agents, including the omegas, must update their positions in line with the best search agent’s position. Therefore, the position of a search agent is updated using the following equations:

7 $$\overrightarrow {D}_{\alpha } = \left| {\overrightarrow {C}_{1} \overrightarrow {X}_{\alpha } - \overrightarrow {X} } \right|,\overrightarrow {D}_{\beta } = \left| {\overrightarrow {C}_{2} \overrightarrow {X}_{\beta } - \overrightarrow {X} } \right|,\overrightarrow {D}_{\delta } = \left| {\overrightarrow {C}_{3} \overrightarrow {X}_{\delta } - \overrightarrow {X} } \right|$$

8 $$\overrightarrow {X}_{1} = \overrightarrow {X}_{\alpha } - \overrightarrow {A}_{1} \overrightarrow {D}_{\alpha } ,\overrightarrow {X}_{2} = \overrightarrow {X}_{\beta } - \overrightarrow {A}_{2} \overrightarrow {D}_{\beta } ,\overrightarrow {X}_{3} = \overrightarrow {X}_{\delta } - \overrightarrow {A}_{3} \overrightarrow {D}_{\delta }$$

9 $$\overrightarrow {X} \text{(t + 1)} = \frac{{\overrightarrow {X}_{1} + \overrightarrow {X}_{2} + \overrightarrow {X}_{3} }}{3}$$

##### Searching for prey and attacking prey

These two operations are ensured by the variation of adaptive values $\overrightarrow {a}$ and $\overrightarrow {A}$, which allow the GWO algorithm to transit smoothly between exploration and exploitation. During the decrease of $\overrightarrow {A}$, and when |A| ≥ 1, one-half of the iterations are intended to exploration (i.e., diverge from the prey), while the remaining half of the iterations are dedicated to exploitation when |A| < 1 (i.e., converge to the prey).

**Figure 3:**
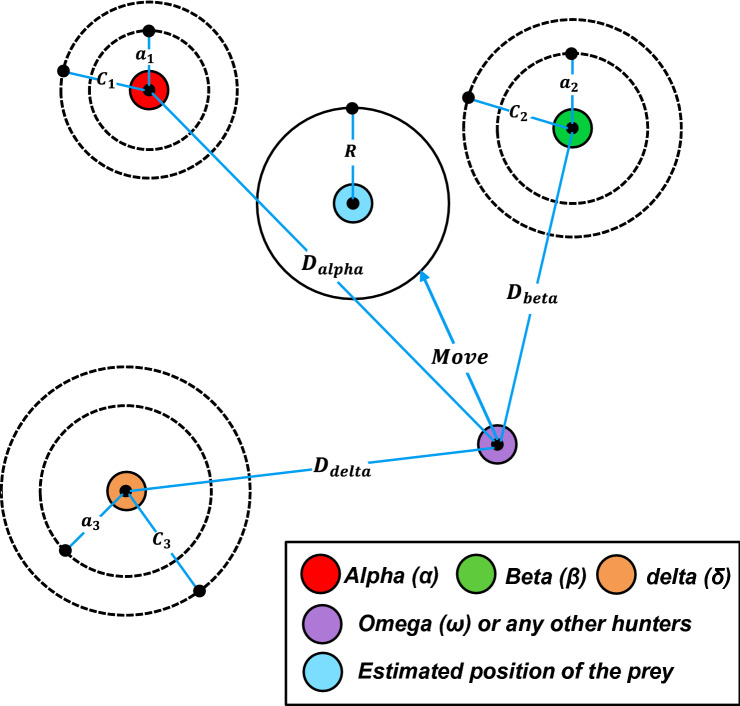
The GWO algorithm methodology.

Based on alpha, beta and delta positions, the methodology used by a search agent (also called a search grey wolf) to update its position in a 2D search space is illustrated in Fig. 3. As shown, the optimal solution would be in a random place inside a circle in the search space, which is determined by alpha, beta, and delta positions. Otherwise, alpha, beta, and delta estimate the prey position (optimal solution), while the remaining wolves update their position at random around the prey^33^.

#### Hybrid GWO–PSO algorithm

Hybrid GWO–PSO is an SI-based optimization algorithm recently developed in 2017 by Narinder Singh et al.^29^. The basic hybridization philosophy of this algorithm is to combine the exploration capability in the GWO algorithm on the one hand, and the exploitation ability in the PSO algorithm on the other hand, to obtain the strength of both variants. For this purpose, the best three search agents' positions (α, β and δ) are updated in the search space by the new equations motioned in (10) instead of the usual equations of (7). In other words, the grey wolves' exploration and exploitation in the search space are controlled by an inertia constant $\left( {w } \right)$ as modelled by the following equations:

10 $$\overrightarrow {D}_{\alpha } = \left| {\overrightarrow {C}_{1} \overrightarrow {X}_{\alpha } - w{ * }\overrightarrow {X} } \right|,\overrightarrow {D}_{\beta } = \left| {\overrightarrow {C}_{2} \overrightarrow {X}_{\beta } - w{ * }\overrightarrow {X} } \right|,\overrightarrow {D}_{\delta } = \left| {\overrightarrow {C}_{3} \overrightarrow {X}_{\delta } - w{ * }\overrightarrow {X} } \right|$$

11 $$\overrightarrow {X}_{1} = \overrightarrow {X}_{\alpha } - \overrightarrow {A}_{1} \overrightarrow {D}_{\alpha } ,\overrightarrow {X}_{2} = \overrightarrow {X}_{\beta } - \overrightarrow {A}_{2} \overrightarrow {D}_{\beta } ,\overrightarrow {X}_{3} = \overrightarrow {X}_{\delta } - \overrightarrow {A}_{3} \overrightarrow {D}_{\delta }$$

On the basis of all the above, the combination of GWO and PSO variants is performed by updating the velocity and positions equations as follows^29^:

12 $$v_{i}^{{k{ + }1}} { = }w { * }\left( {v_{i}^{k} { + }c_{1} r_{1} \left( {X_{1} { - }x_{i}^{k} } \right){ + }c_{2} r_{2} \left( {X_{2} { - }x_{i}^{k} } \right){ + }c_{3} r_{3} \left( {X_{3} { - }x_{i}^{k} } \right)} \right)$$

13 $$x_{i}^{{k{ + }1}} { = }x_{i}^{k} { + } v_{i}^{{k{ + }1}}$$

**Figure 4:**
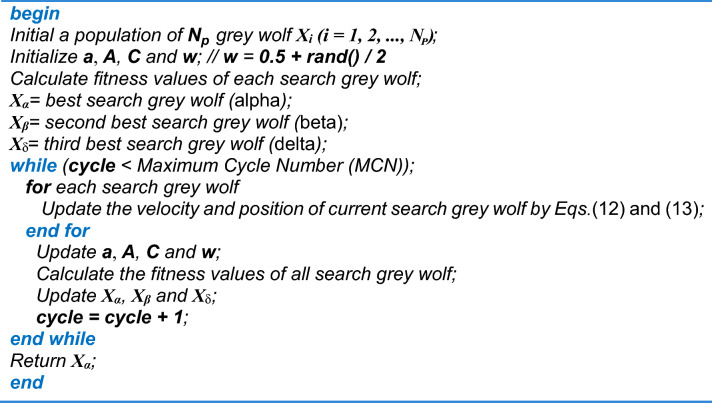
Pseudo-code of hybrid GWO–PSO algorithm.

The hybrid GWO–PSO algorithm can be summarised by the pseudo-code depicted below in Fig. 4.

#### Application of hybrid GWO–PSO toward MPPT

For MPPT realization, a DC–DC power converter is utilized to match the PV array output to the load, where the position of each search agent in the hybrid GWO–PSO algorithm is determined as decision variable, which here represents the duty cycle value ($dc$) of the power converter. Thus, the equations in (10) to (13) are modified to the following:

14 $$D_{\alpha } = \left| {C_{1} {. }dc_{\alpha } - w{ * }dc} \right|,D_{\beta } = \left| {C_{2} {. }dc_{\beta } - w{ * }dc} \right|,D_{\delta } = \left| {C_{3} {. }dc_{\delta } - w{ * }dc} \right|$$

15 $$dc_{1} = dc_{\alpha } - A_{1} D_{\alpha } ,dc_{2} = dc_{\beta } - A_{2} D_{\beta } ,dc_{3} = dc_{\delta } - A_{3} D_{\delta }$$

16 $$v_{i}^{{k{ + }1}} { = }w { * }\left( {v_{i}^{k} { + }c_{1} r_{1} \left( {dc_{1} { - }dc_{i}^{k} } \right){ + }c_{2} r_{2} \left( {dc_{2} { - }dc_{i}^{k} } \right){ + }c_{3} r_{3} \left( {dc_{3} { - }dc_{i}^{k} } \right)} \right)$$

17 $$dc_{i}^{{k{ + }1}} { = }dc_{i}^{k} { + } v_{i}^{{k{ + }1}}$$

The fitness of each search agent (i.e., the duty cycle of the power converter) is selected here as the output power ($Ppv$) of the PV array. So, to assess the duty cycles, a pulse-width-modulation (PWM) signal is generated successively by the digital controller according to the values of these duty cycles. Then, the corresponding PV power ($Ppv_{i}$) of each duty cycle ($dc_{i}$) can be computed from the measured PV voltage ($Vpv_{i}$) and PV current ($Ipv_{i}$). It is important to note that to obtain correct samples, the interval of time between two successive assessments of the duty cycle ($Ts$) must be higher than the settling time of the power converter. Furthermore, to detect if ever a change of the climatic conditions takes place, the following inequality is adopted here:

18 $$\frac{{|Ppv_{new} - Ppv_{last} |}}{{Ppv_{last} }} \ge \Delta Ppv(\% )$$

**Figure 5:**
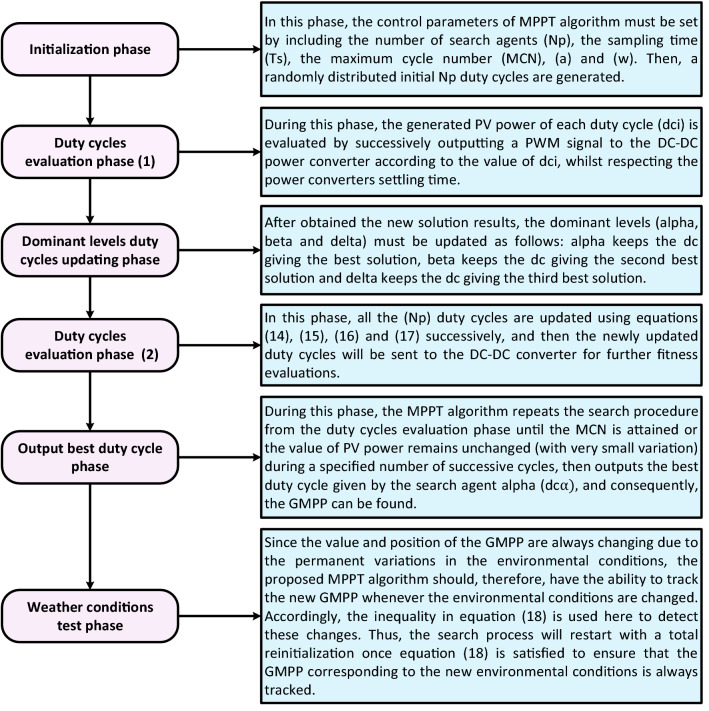
Application procedure of the proposed GWO–PSO based MPPT.

**Figure 6:**
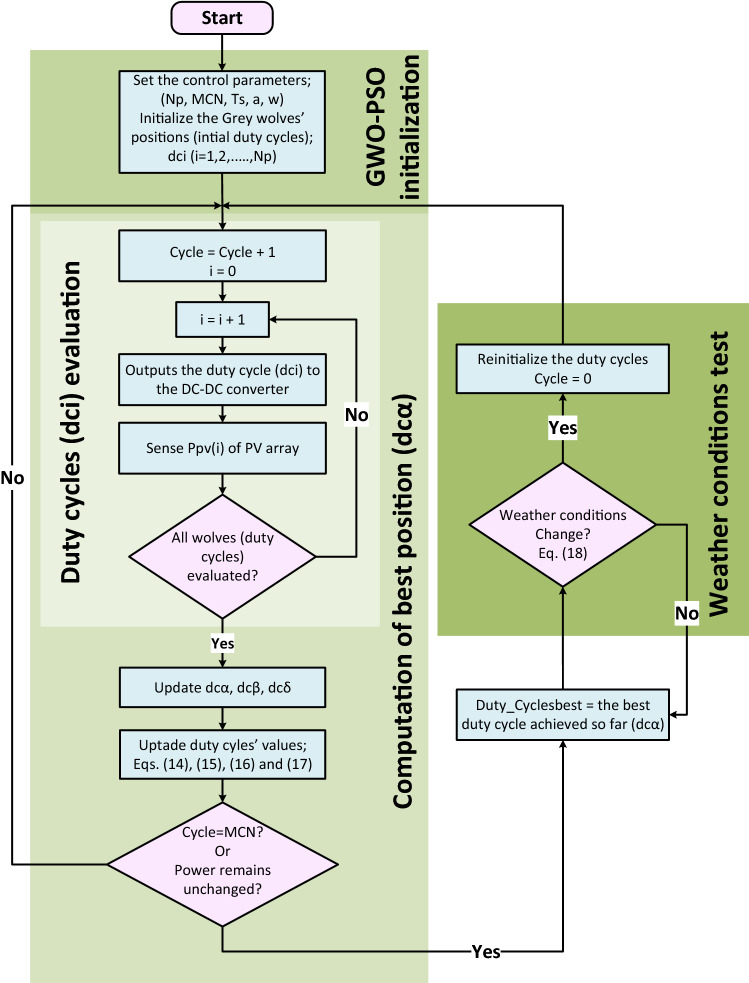
Flowchart of the proposed GWO–PSO based MPPT.

Figure 5 depicts the application procedure of the suggested GWO–PSO-based MPPT and Fig. 6 briefly presents it in flowchart form.

## Implementation and results

### Simulation setup

**Figure 7:**
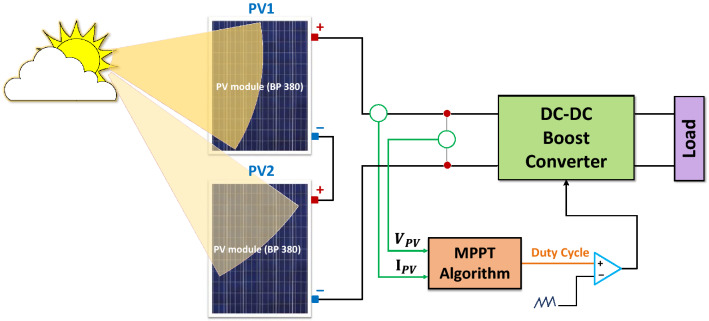
The block diagram used to realize the proposed MPPT method.

The 160 W PV system depicted in Fig. 7 is designed and implemented to test the performance of the proposed hybrid GWO–PSO based MPPT. It consists of two BP 380 PV modules connected in series, a boost-type DC–DC converter, an MPPT controller, and a DC load.

**Figure 8:**
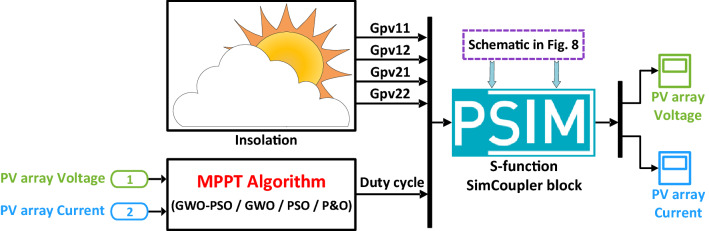
Implemented simulink model for MPPT controller.

**Figure 9:**
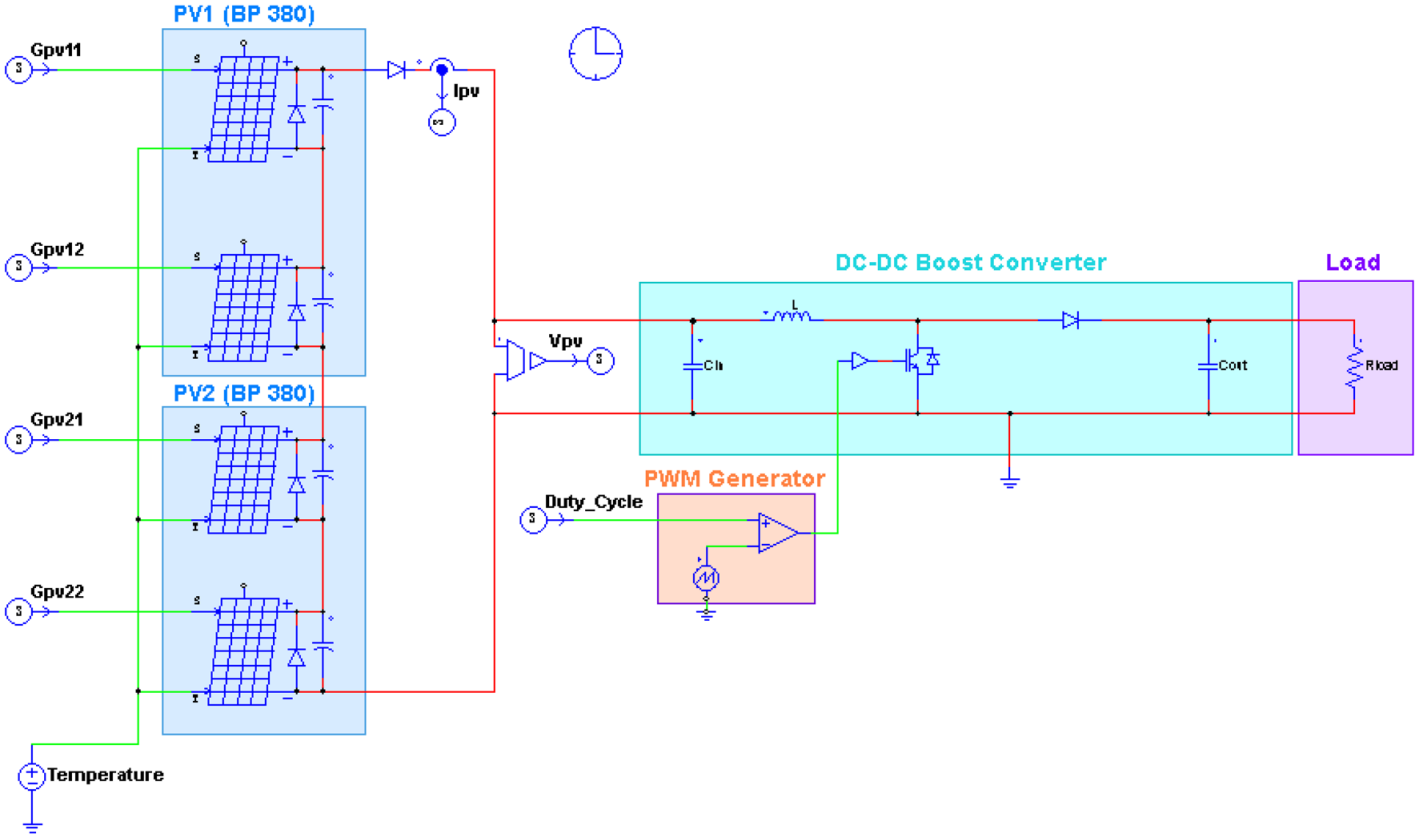
PV array and boost-type DC–DC converter circuits employed in PSIM environment.

In this study, the effectiveness and feasibility of the proposed method are evaluated using a co-simulation technique that combines PSIM and MATLAB/SIMULINK software environments. The physical components, such as the PV modules and the DC–DC boost converter, are modelled in PSIM, while the MPPT algorithm is implemented in MATLAB/SIMULINK. In addition, a comparative performance evaluation of the proposed GWO–PSO algorithm against those of the GWO, PSO and P&O based MPPT reported in^22,26^ and^34^ respectively, under dynamic environmental operating conditions, is also performed. Figure 8 illustrates the MATLAB/SIMULINK model employed to implement the MPPT controller, while the PSIM circuit used to implement the physical parts of the system is depicted in Fig. 9, where two bypass diodes are considered to protect eighteen cells in each BP 380 PV module. The synchronization between MATLAB/SIMULINK and PSIM software environments is ensured by the SimCoupler block, as illustrated in Fig. 8. Table 3 lists the critical parameters of the developed MPPT techniques, namely hybrid GWO–PSO, GWO, PSO, and P&O.

**Table 3:** Parameters of GWO–PSO, GWO, PSO, and P&O based MPPT methods.

GWO–PSO		GWO		PSO		P&O	
*T*_*s*_	0.05	*T*_*s*_	0.05	*T*_*s*_	0.05	*T*_*s*_	0.01
∆*P*_*pv*_	2%	∆*P*_*pv*_	2%	∆*P*_*pv*_	2%	∆*P*_*pv*_	2%
*Np*	*6*	*Np*	6	*Np*	6	∆*dc*	0.01
*MCN*	30	*MCN*	30	*MCN*	30	–	–
a	2	a	2	w	0.4	–	–
w	${0}{.5 + rand()/2}$	c	2	C1	1.6	–	–
–	–	–	–	C2	1.2	–	–

### Results and discussion

#### Global MPP tracking test

To examine the capability of the hybrid GWO–PSO based metaheuristic MPPT algorithm to track the GMPP, an in-depth simulation study was carried out under both stable and transient SPs. And as it is very difficult to test all inhomogeneous insolation conditions, the four SPs plotted in Fig. 2 were considered and used in this work. As illustrated in Fig. 2, the preselected SPs include one uniform insolation pattern (SP1) and three non-uniform insolation patterns (SP2, SP3 and SP4), allowing the proposed MPPT algorithm to be tested under uniform insolation and PSCs.

**Figure 10:**
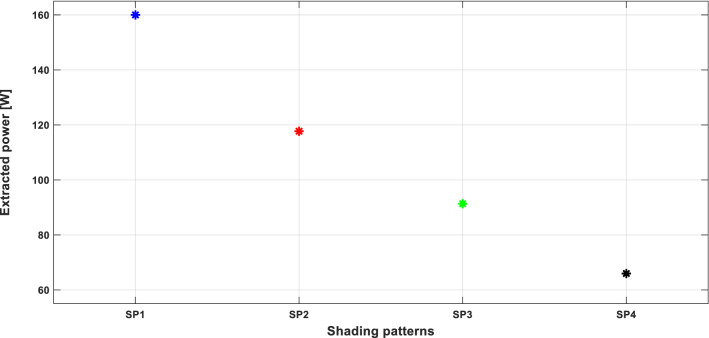
PV power extracted using the hybrid GWO–PSO based MPPT for the four different SPs.

First, a test under stable SPs is performed. Figure 10 depicts the output power distribution of the PV array using the hybrid GWO–PSO based MPPT method under the aforementioned four SPs. Where, for each SP, the MPPT algorithm was executed 100 times to make the results reliable and trusted. As seen in Fig. 10, the output PV power distribution is located around the corresponding GMPP value for each SP, demonstrating that the GWO–PSO based MPPT method can successfully track the GMPP under both uniform insolation and PSCs. Moreover, it can be concluded from the results obtained that the convergence ability of the proposed MPPT algorithm is independent of the initial conditions of the search process.

Since the PV array’s output power varies with the environmental conditions, which are usually dynamic, the value and position of the GMPP are therefore constantly changing. Thus, the proposed MPPT algorithm must be capable of tracking the new GMPP under varying SPs. Toward this end, the following three test cases were used to perform a second test under transient SPs:

- Test 1: SP transits from SP1 to SP3 at t = 15 s.
- Test 2: SP transits from SP3 to SP2 at t = 30 s.
- Test 3: SP transits from SP2 to SP4 at t = 45 s.

**Figure 11:**
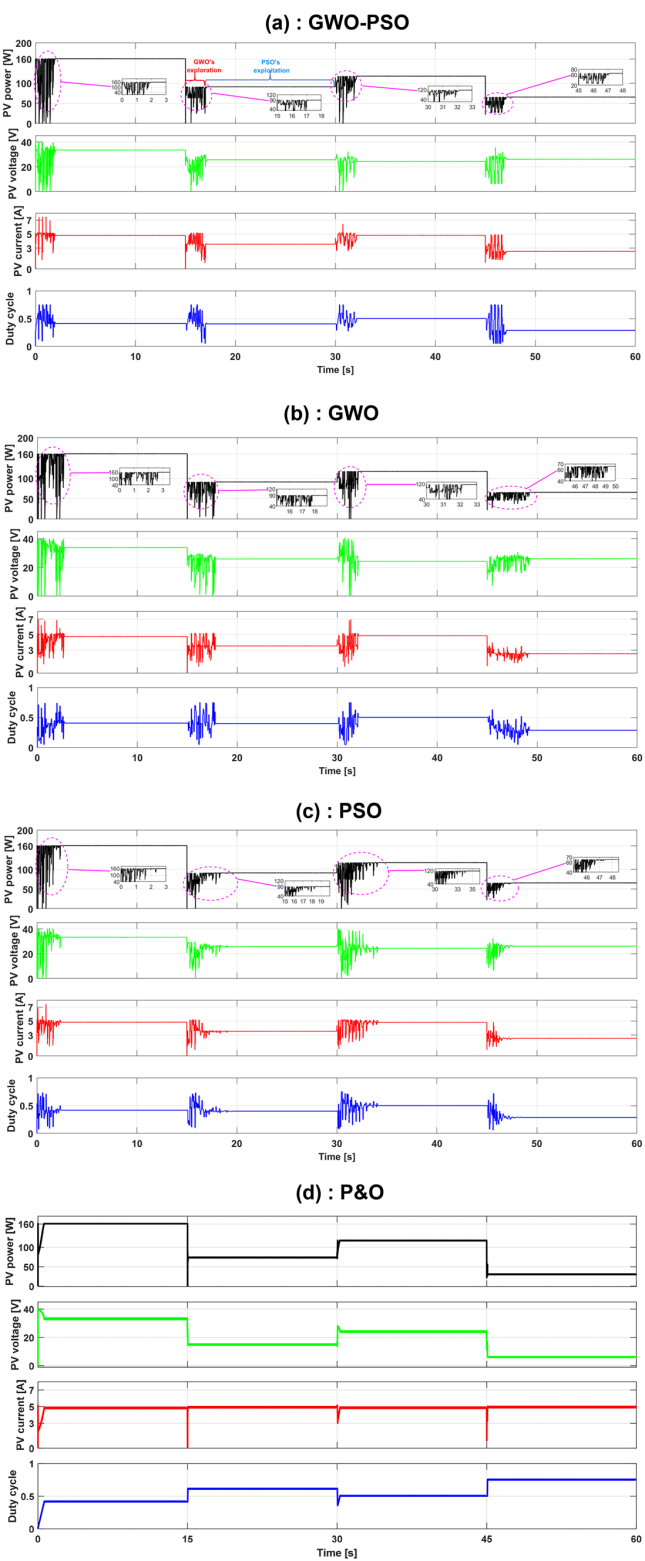
Obtained curves under the different shading pattern variations using: (**a**) GWO–PSO, (**b**) GWO, (**c**) PSO, (**d**) P&O based MPPT methods.

The resulting tracking curves are given in Fig. 11a, which illustrates the dynamic responses of PV power, voltage, and current, and the corresponding duty cycle for each test. As indicated in Fig. 11a, the proposed hybrid GWO–PSO based MPPT successfully converges to the GMPP corresponding to pattern SP1 at first. And when the SP moves from a homogeneous insolation (SP1) to inhomogeneous insolations, such as SP3, SP2 and SP4 at times 15 s, 30 s and 45 s respectively, the proposed MPPT algorithm detects these changes using Eq. (18) and thus restarts the search with a total reinitialization, which allows it to successfully track again all GMPPs corresponding to the new environmental conditions.

#### Comparative performance assessment

This section presents a comparative performance assessment of the proposed hybrid GWO–PSO based MPPT against those of famous existing MPPT algorithms, namely: GWO, PSO and P&O. The dynamic responses of the GWO-, PSO-, and P&O-based MPPT algorithms under the same three previous test scenarios are shown in Fig. 11b–d respectively. From Fig. 11, it can be observed that the metaheuristic algorithms (i.e., GWO–PSO, GWO and PSO) successfully converge to the GMPP corresponding to the different SPs with a noticeable superiority of the GWO–PSO concerning GMPP tracking speed. While the P&O algorithm fails to differentiate between GMPP and LMPP under PSCs and consequently converges to the MPP that comes in contact first, which may be LMPP (in the case of SP3 and SP4) or GMPP (in the case of SP2). For further investigation, a comparison was made based on the three performance indices presented in Table 4, where all algorithms were executed 100 times for each SP provided in Fig. 2. As seen in Table 4, the hybrid GWO–PSO based MPPT method shows great superiority over GWO-, PSO- and P&O-based MPPT methods in terms of accuracy, GMPP tracking speed, and efficiency. Moreover, a qualitative comparison of the proposed GWO–PSO based MPPT versus different MPPT methods existing in the literature was also carried out and reported in Table 5. It can be inferred from Table 5 that the hybrid GWO–PSO based MPPT method outperforms all other MPPT methods, and thus it can be considered as an effective solution for handling partial shading in PV systems since it requires only two control parameters to achieve very high efficiency, and its convergence to GMPP is independent of the search process's initial conditions.

**Table 4:** Comparison between the GWO–PSO, gwo, pso and p&o based mppt methods under different shading patterns.

Pattern no.	Tracking algorithm	Mean power (W)	Tracking speed (s)	Optimal power (W)	Efficiency (%)
SP1	GWO–PSO	159.95	1.95	160	99.97
	GWO	159.93	2.75		99.96
	PSO	159.92	2.63		99.95
	P&O	159.84	1.01		99.90
SP2	GWO–PSO	117,59	2.11	117.7	99.90
	GWO	117.41	2.23		99.75
	PSO	116.92	4.26		99.33
	P&O	116.74	0.74		99.18
SP3	GWO–PSO	91.05	2.05	91.35	99.67
	GWO	90.97	2.87		99.58
	PSO	90.19	3.57		98.73
	P&O	74.04	0.53		81.05
SP4	GWO–PSO	65.71	2.08	65.96	99.13
	GWO	65.39	4.32		99.61
	PSO	65.11	2.59		98.71
	P&O	31.18	0.51		47.27

**Table 5:** Comparison of the GWO–PSO-based mppt with other mppt techniques on a qualitative basis.

MPPT algorithms	P&O	PSO	GWO	ABC^24^	GWO-FLC^28^	Proposed
Tracking speed	Varies	Medium	Medium	Medium	Medium	Fast
Steady-state power oscillation	Large	Zero	Zero	Zero	Zero	Zero
Power efficiency	High for uniform irradiation Low for non-uniform irradiation	High	High	High	High	High
Tracking accuracy	Low	Good	High	High	High	High
Number of control parameters	1	3	2	2	6	2
Convergence to GMPP	LMPP or GMPP, depending on which one comes in contact first	Yes	Yes	Yes	Yes	Yes
Initial conditions dependent	Yes	Heavily depends on the agents' initial position	No	No	Heavily depends on FLC design parameters	No
Implementation complexity	Low	Medium	Medium	Medium	High	Medium

## Conclusion

This paper presents and discusses a new MPPT controller based on the hybrid GWO–PSO metaheuristic algorithm for harvesting the maximum available power from a PV array operating under PSCs. The proposed GWO–PSO based MPPT scheme has been given and implemented for a 160 W PV system using MATLAB/SIMULINK and PSIM software environments. In addition, a performance comparison assessment of the suggested MPPT method against famous existing MPPT methods, namely GWO, PSO and P&O, was also performed in this study. The simulation results carried out under different partial shading scenarios show the great superiority of the new hybrid GWO–PSO based MPPT method over other methods (GWO, PSO and P&O) concerning tracking accuracy, convergence speed to GMPP and efficiency. Furthermore, the proposed hybrid algorithm's convergence is independent of the initial conditions of the search process, and it requires only two control parameters, which makes it simpler and more flexible. Plus, it does not need any prior knowledge of PV array characteristics, making it easy to implement in larger PV systems, whether off-grid or on-grid.

In this work, the effectiveness of the proposed MPPT method has been verified using a co-simulation methodology, but the hardware implementation of this method is not done. Therefore, it would be interesting to implement this method in a microcontroller or a DSP (Digital Signal Processor) board. To this end, an experimental setup will be made to examine the GWO–PSO based MPPT method in a real PV system environment. Then, a thorough investigation will also be conducted to implement this hybrid MPPT method in multistring PV array systems operating under PSCs.
